# Therapeutic Strategies for Hepatocellular Carcinoma: Current Advances and Future Perspectives

**DOI:** 10.3390/vaccines14020189

**Published:** 2026-02-18

**Authors:** Palaniyandi Muthukutty, Jeong Heo, So Young Yoo

**Affiliations:** 1 Institute of Nanobio Convergence, Pusan National University, Busan 46241, Republic of Korea; pmkpalani@gmail.com; 2 Department of Internal Medicine, College of Medicine, Pusan National University, Busan 49241, Republic of Korea; jheo@pusan.ac.kr; 3 Medical Research Institute, Pusan National University Hospital, Busan 49241, Republic of Korea

**Keywords:** hepatocellular cancer, tumor microenvironment, immunotherapy, immune checkpoint inhibitors, neoantigens, oncolytic viruses, bispecific antibodies, combination immunotherapy

## Abstract

Hepatocellular carcinoma (HCC) accounts for approximately 90% of primary liver cancers and remains a leading cause of cancer-related mortality worldwide. The management of HCC poses a major therapeutic challenge due to its pronounced molecular heterogeneity, frequent late-stage diagnosis, and intrinsic resistance to both conventional and modern therapeutic modalities. Furthermore, the relatively low tumor mutational burden and the presence of a profoundly immunosuppressive tumor microenvironment (TME) substantially limit the efficacy of immune-based interventions, particularly in advanced disease stages. In recent years, novel immunotherapeutic approaches—including immune checkpoint blockade (ICB), oncolytic virus therapy, and genetically engineered immune cell-based therapies—have garnered significant attention. Nevertheless, durable clinical responses and meaningful improvements in overall survival remain limited, underscoring the complexity of achieving effective immune control in HCC. Emerging evidence suggests that rational combination immunotherapy strategies may offer new therapeutic opportunities by overcoming immune resistance mechanisms. In this review, we provide a comprehensive overview of current therapeutic strategies for HCC, with particular emphasis on immunotherapeutic approaches. We discuss common clinical challenges spanning diagnosis to treatment resistance, critically evaluate key clinical trial outcomes, and highlight future directions aimed at improving therapeutic efficacy and long-term disease control.

## 1. Introduction

Hepatocellular carcinoma (HCC) is a major global health burden and currently ranks as the third leading cause of cancer-related mortality worldwide, accounting for approximately 80–90% of primary liver cancers [1]. The development of HCC is multifactorial, with chronic liver disease—most commonly cirrhosis—serving as the central underlying condition in the majority of cases [2]. Persistent liver injury resulting from chronic viral infections, particularly hepatitis B virus (HBV) and hepatitis C virus (HCV), contributes to nearly 80% of HCC cases. Additional etiological factors include excessive alcohol consumption, non-alcoholic steatohepatitis (NASH), obesity, metabolic disorders such as diabetes, autoimmune hepatitis, exposure to aflatoxins, and inherited genetic predispositions. These insults drive sustained hepatic inflammation, ultimately promoting fibrosis, cirrhosis, and malignant transformation of hepatocytes [3]. The complex interplay between genetic susceptibility and viral or non-viral risk factors critically influences disease severity and progression, culminating in hepatocarcinogenesis [4].

Substantial advances have been made in elucidating the molecular pathophysiology of HCC; however, the translation of these discoveries into effective clinical interventions remains limited [5]. Over the past decade, global efforts in genetic and molecular subtyping of HCC—integrated with clinical, etiological, and histopathological analyses—have improved disease stratification and led to the identification of distinct molecular subclasses [6]. Despite these advances in diagnosis and therapeutic development, the overall prognosis for HCC remains poor, with a five-year survival rate of approximately 20% and recurrence rates approaching 90% even after curative treatment [7,8].

**Figure 1:**
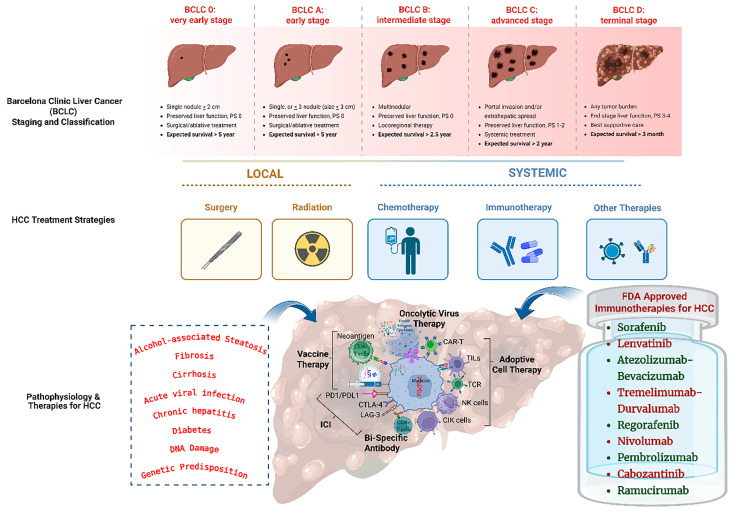
Hepatocellular Carcinoma: Classification, Progression, Treatment strategies, Pathophysiology and approved drugs for the treatment of HCC. (Created in BioRender. Muthukutty, P. (2026) https://BioRender.com/j1e6dqs) (accessed on 27 December 2025). CAR-T: Chimeric Antigen Receptor-T cells; TIL: Tumor-Infiltrating Lymphocytes; TCR: T-Cell Receptor; NK: Natural Killer cells; CIK: Cytokine Induced Killer Cells; PD-1: Programmed Cell Death Protein-1; PD-L1: Programmed Cell Death Ligand Protein-1; CTLA-4: Cytotoxic T-Lymphocyte-associated Antigen 4; LAG-3: Lymphocyte Activation Gene 3.

Clinical management of HCC relies on staging systems that consider tumor burden, liver function, and patient performance status. Among these, the Barcelona Clinic Liver Cancer (BCLC) staging system is the most widely adopted framework for guiding treatment decisions and clinical trial design [9]. According to BCLC classification, early-stage HCC (stages 0 and A) may be managed with curative-intent therapies, including surgical resection, radiofrequency ablation (RFA), or liver transplantation; nevertheless, tumor recurrence remains a significant challenge. Patients with intermediate-stage disease (stage B) are typically treated with transarterial chemoembolization (TACE) or other locoregional therapies, with median survival rarely exceeding 20 months. Advanced-stage HCC (stages C and D) is managed primarily with systemic therapies and best supportive care, where median overall survival remains limited to approximately 7 months [10]; see Figure 1.

Despite progress in surveillance, staging, and treatment modalities, the prognosis for patients with HCC remains dismal, largely due to late-stage diagnosis and the limited efficacy of available therapeutic options [11]. Epidemiological projections estimate that the global incidence of HCC will exceed one million cases annually by 2030, underscoring the urgent need for improved management strategies [12]. While early detection enables potentially curative interventions such as surgical resection, local ablation, or liver transplantation, the majority of patients present with advanced disease, for which locoregional and systemic therapies are largely palliative in nature [13].

Given these limitations, there is a critical need to develop novel therapeutic strategies that extend beyond conventional surgical, locoregional, and systemic approaches [14]. Increasing evidence highlights the role of immune surveillance, immune cell infiltration, and chronic inflammation in hepatocarcinogenesis, suggesting that immunotherapy-based strategies may offer new opportunities to inhibit tumor growth, invasion, and metastasis [15,16]. In this review, we comprehensively examine current immunotherapy-based approaches for HCC, discuss the key clinical and biological challenges limiting their efficacy, and outline future strategies aimed at improving therapeutic outcomes and long-term disease control.

## 2. Immunosuppressive Microenvironment of Liver

The liver possesses unique immunological properties that collectively establish a highly tolerogenic environment, shaped by continuous exposure to dietary antigens, microbial products, and endogenous metabolites derived from the gut–liver axis [17]. This immune tolerance is maintained by specialized non-parenchymal liver-resident cells, including hepatic stellate cells (HSCs), liver sinusoidal endothelial cells (LSECs), and Kupffer cells, which actively regulate immune activation and suppression under physiological conditions [18]. HCC typically arises in the context of chronic liver injury, where persistent inflammation provides a fertile ground for tumor initiation and progression.

Chronic hepatic inflammation induces sustained oxidative stress, leading to the accumulation of reactive oxygen species (ROS) and reactive nitrogen species. These mediators promote chromosomal instability, DNA damage, and epigenetic alterations that collectively drive hepatocarcinogenesis [19]. As HCC evolves from premalignant lesions to advanced disease, the tumor microenvironment (TME) undergoes dynamic remodeling. This complex milieu, composed of malignant hepatocytes, stromal cells, immune cells, and soluble mediators, plays a central role in tumor growth, immune evasion, and therapeutic resistance. Extensive studies have demonstrated that bidirectional interactions between cancer cells and their microenvironment critically shape disease progression by suppressing effective antitumor immune responses through the recruitment and activation of immunosuppressive immune cell populations [20].

Prolonged inflammatory signaling results in extensive immune cell infiltration and tissue remodeling within the liver. Dysregulated cytokine and chemokine networks further amplify oxidative stress and promote fibrosis and cirrhosis, ultimately facilitating malignant transformation [21]. Importantly, the immunological composition of the HCC TME is highly heterogeneous among patients, reflecting differences in disease etiology, genetic background, and tumor stage. The TME is enriched with diverse immune and stromal cell populations whose collective activity determines the balance between immune surveillance and immune suppression.

Based on their functional roles, immune cells infiltrating the HCC TME can be broadly categorized into immunosuppressive and immunostimulatory subsets. Immunosuppressive populations—including myeloid-derived suppressor cells (MDSCs), tumor-associated macrophages (TAMs), and regulatory T cells (Tregs)—are frequently enriched and actively inhibit cytotoxic immune responses [22]. In contrast, immunostimulatory cells such as cytotoxic CD8^+^ T lymphocytes, pro-inflammatory CD4^+^ T helper 1 (Th1) cells, and natural killer (NK) cells mediate antitumor immunity through direct cytotoxicity and cytokine production [23,24]. In HCC, however, the suppressive arm typically dominates, resulting in impaired effector cell function and reduced antitumor activity.

Effective anticancer immune surveillance relies on coordinated interactions between the innate and adaptive immune systems [25]. In HCC, multiple mechanisms converge to impair immune recognition and tumor elimination. These include epigenetic and post-transcriptional silencing of tumor-associated antigens (TAAs), defects in antigen processing and presentation machinery, and the accumulation of immunoregulatory cell populations such as Tregs, inhibitory B cells, MDSCs, and M2-polarized TAMs. In addition, the upregulation of co-inhibitory immune checkpoint pathways, increased expression of tolerogenic enzymes such as arginase-1 and indoleamine 2,3-dioxygenase 1 (IDO1), and reduced immunoglobulin-mediated opsonization further reinforce immune tolerance within the TME [26]. The Wnt/β-catenin signaling pathway plays a critical role in maintaining liver metabolic homeostasis, regeneration, and tissue integrity. Aberrant activation of this pathway is frequently implicated in hepatocarcinogenesis and contributes to HCC progression. Importantly, Wnt/β-catenin activation alone is often insufficient to drive full malignant transformation and may cooperate with additional oncogenic alterations (e.g., MET activation or glypican-3–associated signaling). Therefore, therapeutic strategies that concurrently target key oncogenic drivers and attenuate Wnt/β-catenin signaling may enhance tumor control and help overcome resistance to therapy.

Despite this immunosuppressive environment, spontaneous tumor-specific T-cell responses can be detected in patients with HCC. CD8^+^ T cells recognizing alpha-fetoprotein (AFP), glypican-3 (GPC3), melanoma-associated antigen-1 (MAGE-1), and New York esophageal squamous cell carcinoma 1 (NY-ESO-1) have been identified in both peripheral blood and tumor tissues, and their presence has been associated with improved patient survival [27]. However, the functional efficacy of these effector T cells is frequently compromised by the suppressive TME and elevated expression of immune checkpoint molecules, ultimately facilitating tumor immune evasion [28].

Collectively, these findings highlight the central role of the immunosuppressive hepatic TME in limiting the efficacy of antitumor immune responses in HCC. Accordingly, ongoing clinical trials are increasingly focused on therapeutic strategies that simultaneously inhibit pro-tumorigenic pathways and enhance immune-mediated cytotoxicity, with the goal of reprogramming the TME to support durable antitumor immunity [29].

## 3. Immunotherapies for HCC

Immunotherapy has emerged as a transformative therapeutic modality in oncology, particularly through the development of immune checkpoint inhibitors (ICIs), which have demonstrated remarkable clinical benefit across multiple solid malignancies [30]. In hepatocellular carcinoma, immunotherapeutic approaches have attracted considerable interest due to their ability to extend overall survival (OS) while maintaining manageable toxicity profiles [31]. See Figure 1. Unlike conventional cytotoxic therapies, immunotherapy functions by relieving tumor-induced immune suppression, thereby restoring endogenous antitumor immune responses [32].

By enhancing cellular immunity, regulating immune activation thresholds, and reinvigorating T-cell effector function, immunotherapy can effectively limit tumor growth and progression [33]. Among these approaches, ICIs—monoclonal antibodies that target key inhibitory pathways within the cancer–immunity cycle—have reshaped the systemic treatment paradigm for advanced malignancies. The most extensively studied immune checkpoints include programmed cell death protein 1 (PD-1), its ligand PD-L1, and cytotoxic T-lymphocyte–associated protein 4 (CTLA-4). While PD-1 engagement by PD-L1 or PD-L2 suppresses T-cell activity in peripheral tissues, CTLA-4 competitively inhibits the binding of CD28 to B7 ligands, thereby attenuating T-cell priming during early immune activation [34].

Overexpression of PD-L1 has been documented in the TME of numerous solid tumors, including esophageal, colorectal, pancreatic, gastric, lung, breast cancers, as well as HCC [35]. In patients with HCC, therapeutic targeting of one or more immune checkpoint pathways has the potential to promote tumor regression by restoring T-cell–mediated antitumor immunity. Immune checkpoint inhibitors—monoclonal antibodies that selectively block PD-1, PD-L1, and CTLA-4—function by relieving inhibitory signaling and reactivating effector T-cell responses [36]. HCC is therefore considered a particularly relevant candidate for immune-based therapeutic strategies, given its strong association with chronic inflammation and immune-mediated disease pathogenesis [37,38].

Nonetheless, the complex and multifaceted nature of the HCC TME reveals the presence of multiple, non-redundant immunosuppressive pathways that collectively impose a substantial barrier to effective immunotherapy. Early evidence from single-arm clinical studies has demonstrated signs of antitumor immune reconstitution following ICI treatment in HCC, generating initial clinical interest and proof of biological activity [39]. As immunotherapy continues to emerge as a key component of the therapeutic landscape for HCC, a deeper understanding of the immune-biological mechanisms underlying HCC progression and treatment resistance is essential. In the following sections, we discuss the rationale for the development of immune-based therapies in liver cancer and highlight key immunological pathways that may be exploited to enhance therapeutic efficacy.

### 3.1. Immune Checkpoint Inhibitors

Immune checkpoint inhibitors exert antitumor effects by disrupting inhibitory signaling pathways on T cells or antigen-presenting cells, thereby facilitating immune recognition and elimination of malignant cells. Tumors characterized by a high tumor mutational burden typically exhibit superior responsiveness to ICIs due to increased neoantigen availability [40]. In contrast, HCC generally exhibits a relatively low mutational burden, which partially explains its modest response rates to checkpoint inhibition.

Immune checkpoint molecules are expressed across a broad range of immune cell populations, including T cells, B cells, dendritic cells (DCs), natural killer (NK) cells, monocytes, tumor-associated macrophages (TAMs), and myeloid-derived suppressor cells (MDSCs). Among these checkpoints, PD-1/PD-L1 and CTLA-4 have been most extensively evaluated and currently constitute the backbone of immunotherapy in HCC. PD-1 is expressed on activated CD8^+^ and CD4^+^ T cells, regulatory T cells (Tregs), B cells, NK cells, and DCs, underscoring its central role in immune regulation [41].

PD-1 has emerged as a particularly attractive immunotherapeutic target due to its ability to attenuate effector T-cell responses upon ligand engagement [42]. In single-arm phase II clinical trials, the PD-1 inhibitors nivolumab and pembrolizumab demonstrated clinically meaningful activity as second-line therapies in patients with advanced HCC who were refractory or intolerant to sorafenib. Objective response rates of approximately 15–20%, including complete responses in a minority of patients, were observed and correlated with prolonged survival outcomes. In the CheckMate 040 trial, patients treated with nivolumab achieved a median duration of response of 17 months (95% CI, 6–24 months), with more than 80% of responders surviving beyond two years [43].

Similarly, the phase III KEYNOTE-240 trial evaluating pembrolizumab versus placebo in patients previously treated with sorafenib demonstrated a trend toward improved overall survival (hazard ratio, 0.78; *p* = 0.023), although the predefined statistical significance threshold was not met. Notably, a subset of patients derived durable clinical benefit, with nearly 20% remaining progression-free for over one year, compared with less than 7% in the placebo arm. Based on these data, nivolumab and pembrolizumab received regulatory approval as second-line treatment options for advanced HCC [44].

CTLA-4 represents another critical immune checkpoint that modulates T-cell activation by counterbalancing co-stimulatory signaling. By antagonizing CD28-mediated activation through competition for CD80 and CD86 binding, CTLA-4 limits excessive immune activation and promotes immune tolerance. Consequently, CTLA-4 blockade has been explored as a therapeutic strategy in advanced HCC. Several phase I and II clinical trials have demonstrated encouraging antitumor activity, while ongoing studies continue to refine its clinical utility [45,46].

In a phase II study, the anti–CTLA-4 antibody tremelimumab achieved a partial response rate of 17.6%, a median time to progression of 6.48 months, and an acceptable safety profile. Furthermore, ipilimumab—another CTLA-4–targeting antibody—has demonstrated enhanced antitumor efficacy when administered in combination regimens compared with monotherapy [47]. Additional immune checkpoint inhibitors targeting PD-L1, such as durvalumab and avelumab, as well as CTLA-4 inhibitors including tremelimumab and ipilimumab, have been evaluated in clinical studies. Nevertheless, only approximately 20% of patients exhibit objective responses to single-agent immune checkpoint blockade [48,49].

These limitations have driven the development of combination strategies designed to amplify antitumor immunity. Approaches integrating multiple ICIs or combining ICIs with other therapeutic modalities have demonstrated improved clinical outcomes [50]. Notably, the combination of tremelimumab plus durvalumab received orphan drug designation and, in a phase III trial, was associated with improved overall survival and a manageable safety profile. Median overall survival was 16.43 months (95% confidence interval [CI], 14.16–19.58) with STRIDE, 16.56 months (95% CI, 14.06–19.12) with durvalumab, and 13.77 months (95% CI, 12.25–16.13) with sorafenib. The HIMALAYA study established this regimen as a first-line treatment option for advanced HCC, surpassing sorafenib in efficacy [51]. Additionally, the combination of ipilimumab and nivolumab has received accelerated regulatory approval as a second-line therapy for patients with sorafenib-refractory HCC [20]. In the phase III LEAP-002 trial (NCT03713593), lenvatinib plus pembrolizumab did not meet the prespecified co-primary endpoints at the final analysis, although a numerical improvement in overall survival (OS) was observed (median OS, 21.2 vs. 19.0 months; HR 0.84, 95% CI 0.71–1.00) with a manageable safety profile. With longer follow-up, OS remained numerically favorable (median OS, 21.1 vs. 19.0 months; HR 0.80, 95% CI 0.69–0.94).

A summary of completed and ongoing clinical trials evaluating immune checkpoint inhibitors in HCC is provided in Table 1 and Table 2.

Beyond PD-1/PD-L1 and CTLA-4, emerging immune checkpoints such as lymphocyte activation gene 3 (LAG-3), T-cell immunoglobulin and mucin-domain containing protein 3 (TIM-3), and immune receptor tyrosine-based inhibitory motif–containing receptors have garnered increasing interest. In addition, bispecific antibodies targeting multiple immune checkpoints represent a promising next-generation immunotherapeutic strategy [100,101]. Despite these advances, only a minority of patients derive sustained clinical benefit from immune checkpoint inhibition, and treatment failure is frequently associated with tumor progression, hepatic dysfunction, and unfavorable prognosis [102,103].

A major obstacle to optimizing immune checkpoint therapy in HCC is the absence of robust predictive biomarkers capable of accurately identifying responsive patient subsets [104]. Additional mechanisms contributing to treatment resistance include insufficient T-cell infiltration, compensatory upregulation of alternative inhibitory pathways, tumor-intrinsic loss of immunogenicity, alterations in the gut microbiota, and persistent immunosuppressive features of the TME. Collectively, these factors account for the limited response rates observed with immune checkpoint inhibitors in HCC and underscore the need for rational combination strategies and biomarker-guided therapeutic approaches [105].

### 3.2. Adoptive Cell Therapies (ACT)

Adoptive cell therapy (ACT) represents a personalized immunotherapeutic strategy designed to eradicate cancer cells through the ex vivo expansion and reinfusion of autologous or allogeneic immune effector cells. This approach typically involves the isolation of immune cells from a patient’s tumor tissue or peripheral blood, followed by expansion and, in some cases, genetic modification before reinfusion into the patient [106,107]. By introducing large numbers of tumor-reactive immune cells, ACT aims to generate de novo or enhanced antitumor immune responses, underscoring its high specificity and degree of customization.

Several ACT modalities have demonstrated promising anticancer activity in HCC across preclinical and early clinical studies. These include tumor-infiltrating lymphocytes (TILs), cytokine-induced killer (CIK) cells, chimeric antigen receptor (CAR) T cells, natural killer (NK) cells, and T-cell receptor (TCR)–engineered T cells (Table 2) [108]. Despite their conceptual appeal, the clinical translation of ACT in HCC has been constrained by tumor heterogeneity, limited immune cell infiltration, and the profoundly immunosuppressive TME.

#### 3.2.1. Tumor-Infiltrating Lymphocytes (TILs)

TILs constitute a heterogeneous population of immune cells, predominantly composed of effector T lymphocytes that have migrated into tumor tissues. In HCC, TILs are typically present at relatively low frequencies and often exhibit functional impairment, both of which have been associated with unfavorable clinical outcomes [109]. These characteristics distinguish HCC from tumors such as melanoma, where TIL-based therapies have demonstrated substantial clinical success [110].

The generation of TIL-based therapies involves isolating lymphocytes from resected tumor specimens, selecting tumor-reactive T cells, and expanding them ex vivo in the presence of anti-CD3 antibodies and interleukin-2 (IL-2). Following expansion, TILs are reinfused into patients who have undergone lymphodepleting chemotherapy to enhance engraftment and persistence [111]. High-dose IL-2 administration is often required post-infusion to support T-cell survival and proliferation, although this regimen is associated with significant toxicity [112]. Tumor recognition by TILs is mediated through endogenous T-cell receptors (TCRs), which recognize tumor-derived peptide antigens presented by major histocompatibility complex (MHC) molecules on cancer cells, thereby triggering antigen-specific immune activation [113].

Early-phase clinical studies have demonstrated the feasibility and safety of TIL therapy in HCC. In a phase I trial, autologous TILs were successfully expanded and reinfused into patients with minimal treatment-related adverse effects [114]. However, multiple biological and technical barriers have limited the broader application of TIL-based therapies in HCC. These include the low abundance of TILs within HCC tumors, inefficient cell isolation and expansion, functional exhaustion characterized by the upregulation of immune checkpoint molecules such as PD-1, and the highly immunosuppressive hepatic TME [115,116]. In addition, patient tolerance to lymphodepleting conditioning regimens and the systemic toxicity associated with high-dose IL-2 further complicate clinical implementation.

Owing to these limitations, alternative ACT strategies utilizing peripheral blood–derived lymphocytes as a more accessible and scalable source of effector T cells have been increasingly explored for the treatment of HCC [117]. These approaches aim to circumvent the logistical challenges of TIL isolation while enabling genetic engineering strategies to enhance tumor specificity and functional persistence.

#### 3.2.2. Genetically Modified T Cells

The development of genetically modified T-cell therapies, including chimeric antigen receptor (CAR) T cells and T-cell receptor (TCR)–engineered T cells, has generated substantial interest as a next-generation immunotherapeutic strategy for cancer treatment [118]. These approaches typically begin with leukapheresis to collect peripheral blood mononuclear cells, followed by genetic modification using viral or non-viral gene delivery systems. In CAR-T cell therapy, T cells are engineered to express synthetic antibody-based receptors, whereas TCR-engineered T cells are modified to express exogenous tumor-specific TCRs. Through the introduction of CARs or TCRs, T-cell specificity can be reprogrammed to selectively recognize tumor-associated antigens [119].

##### a. T-Cell Receptor (TCR) Engineered T Cell Therapy

TCRs are heterodimeric proteins composed of α and β chains that recognize tumor-derived peptide antigens presented by major histocompatibility complex (MHC) molecules. In TCR-engineered T-cell therapy, tumor-specific TCRs are identified, isolated, and cloned into gene transfer vectors. Autologous T cells obtained from peripheral blood are then transduced to express high-affinity tumor-specific TCRs and subsequently expanded ex vivo prior to reinfusion into patients [120,121]. A critical determinant of therapeutic success in this approach is the identification of appropriate tumor-associated antigens (TAAs).

Several TAAs have been investigated as potential targets for TCR-engineered T-cell therapy in HCC, including AFP, GPC3, MAGE-A1, HBsAg, hTERT, and NY-ESO-1 [122]. These antigens have been evaluated across multiple preclinical studies and early-phase clinical trials. Notably, AFP-specific and HBV-specific TCR-engineered T cells have been explored in patients with HBV-associated HCC, including those treated following liver transplantation or locoregional therapies [123]. Additional trials targeting GPC3, NY-ESO-1, and MAGE-A1 have also been conducted in patients with advanced or relapsed HCC [124].

In a recent phase I clinical trial, AFP-specific TCR-engineered T-cell therapy demonstrated an objective response rate of 9.5% and durable disease stabilization for at least 16 weeks, with manageable safety profiles [125]. Despite these encouraging results, several challenges limit the widespread clinical application of TCR-based therapies. These include mispairing between endogenous and transduced TCR α and β chains, which may generate autoreactive T cells; limited availability of well-defined and tumor-specific antigens; on-target off-tumor toxicity due to low-level antigen expression in normal tissues; off-target cross-reactivity; cytokine release syndrome (CRS); T-cell exhaustion; and restricted expansion and persistence within the immunosuppressive tumor microenvironment (TME) [126,127,128].

Moreover, TCR-engineered T cells are restricted to recognizing antigens presented on MHC molecules. As many tumors, including HCC, downregulate MHC expression to evade immune surveillance, this represents a fundamental limitation of TCR-based strategies [129]. These constraints have motivated the development of alternative approaches, such as CAR-T cell therapy, which enables antigen recognition in an MHC-independent manner [130].

##### b. CAR-T Cell Therapy

CAR-T cell therapy involves genetic modification of T cells to express chimeric antigen receptors capable of recognizing tumor surface antigens independently of MHC presentation. A CAR typically consists of an extracellular single-chain variable fragment (scFv) derived from a monoclonal antibody, a hinge region, a transmembrane domain, and an intracellular signaling domain containing CD3ζ with one or more costimulatory signaling motifs [131].

The evolution of CAR-T cell technology has progressed through multiple generations. First-generation CARs contained only the CD3ζ signaling domain and exhibited limited persistence and efficacy. Second-generation CARs incorporated an additional costimulatory domain, such as CD28 or 4-1BB, enhancing T-cell proliferation and cytotoxicity. Third-generation CARs included multiple costimulatory domains to further improve T-cell survival. Fourth-generation CAR-T cells, often referred to as “armored CARs,” were engineered to secrete transgenic cytokines such as interleukin-12 to enhance antitumor activity within the TME [132]. More recently, fifth-generation CAR-T cells have been developed by incorporating elements of the interleukin-2 receptor β chain and STAT3/5 signaling domains, enabling cytokine signaling upon antigen engagement [133].

Preclinical studies have demonstrated promising antitumor activity of CAR-T cells targeting HCC-associated antigens. Among these, GPC3 has emerged as one of the most extensively investigated targets due to its high expression in HCC and limited expression in normal tissues [134]. Early-phase clinical studies of second-generation glypican-3 (GPC3)–targeted CAR-T cells (NCT02395250, NCT03146234) have reported generally acceptable safety profiles and preliminary, early signals of antitumor activity; however, conclusions remain limited by small cohort sizes and the non-randomized nature of these trials. Additional studies assessing fourth-generation CAR-T cells (NCT03980288, NCT06198296) are currently ongoing. Other CAR-T targets under investigation include AFP, mucin 1 (MUC1), and epithelial cell adhesion molecule (EpCAM) across multiple early-phase clinical trials.

Despite these advances, CAR-T cell therapy in HCC faces challenges similar to those encountered with TCR-based approaches. Tumor heterogeneity, lack of truly tumor-specific antigens, limited infiltration and persistence of CAR-T cells within solid tumors, off-target toxicity, and the immunosuppressive TME remain major obstacles. Furthermore, the complexity, cost, and logistical demands associated with CAR-T cell manufacturing significantly limit widespread clinical implementation. To address these challenges, the identification of neoantigens and the development of allogeneic “off-the-shelf” CAR-T products derived from healthy donors are being actively explored to enhance accessibility and reduce cost [135].

#### 3.2.3. Natural Killer (NK) Cell-Based Therapies

NK cells are innate immune lymphocytes characterized by the absence of CD3 and the expression of CD56. Their cytotoxic activity is governed by a balance between activating receptors, such as natural cytotoxicity receptors (NCRs) and NKG2D, and inhibitory receptors, including killer-cell immunoglobulin-like receptors (KIRs) and NKG2A. Through a mechanism known as “missing-self” recognition, NK cells can eliminate target cells with reduced MHC class I expression without prior antigen sensitization [136].

NK cells can be isolated from peripheral blood mononuclear cells and expanded ex vivo using cytokines such as IL-2, IL-15, IL-12, IL-18, and IL-21 or feeder cell systems before reinfusion following lymphodepleting chemotherapy [137]. Preclinical and clinical studies have demonstrated the therapeutic potential of ex vivo–expanded NK cells; however, their clinical efficacy remains limited. Autologous NK cells often exhibit functional impairment due to inhibitory signaling mediated by self-MHC interactions, exhaustion, and immunosuppressive influences within the TME [138].

To overcome these limitations, alternative sources of NK cells—including allogeneic peripheral blood, umbilical cord blood, and NK cell lines such as NK-92—have been explored [139]. Genetic engineering approaches, including CAR-NK and TCR-NK cells, have also been developed. Compared with CAR-T cells, CAR-NK cells offer potential safety advantages due to their shorter lifespan, reduced risk of graft-versus-host disease, and lower incidence of cytokine-mediated toxicities [140].

Several clinical trials have evaluated NK cell-based therapies in HCC. In a phase II study (MG4101; NCT02008929), patients receiving ex vivo–expanded allogeneic NK cells following hepatic resection exhibited recurrence despite treatment. Phase I and II studies involving NK cells derived from donor liver graft perfusates or expanded ex vivo after curative resection demonstrated favorable safety profiles but limited efficacy in preventing recurrence [86,87]. Persistent challenges include poor tumor infiltration, immunosuppressive TME-mediated inhibition, and the technical complexity of large-scale NK cell expansion [141,142].

#### 3.2.4. Cytokine Induced Killer (CIK) Cells

Cytokine-induced killer (CIK) cells represent a heterogeneous population of immune effector cells characterized by rapid proliferation, non-MHC–restricted cytotoxicity, and limited toxicity toward normal tissues [143]. CIK cells include CD3^+^CD56^+^ NK-like T cells, CD3^+^CD56^−^ cytotoxic T lymphocytes, and CD3^−^CD56^+^ NK cells [144]. These cells are generated ex vivo from patient-derived peripheral blood mononuclear cells using interferon-γ (IFN-γ), interleukin-2 (IL-2), and anti-CD3 monoclonal antibodies.

CIK cells exert antitumor activity through dual mechanisms: cytokine secretion, including IFN-γ, IL-2, and tumor necrosis factor-α (TNF-α), and direct cytotoxicity mediated by activating receptors such as NKG2D, NKp30, and DNAM-1 [145,146,147]. Clinical studies have demonstrated that CIK cell therapy is safe and can improve recurrence-free survival (RFS) and overall survival (OS) in patients with HCC, particularly when administered as adjuvant therapy following curative resection [89,148].

Immuncell-LC, an autologous CIK cell product approved by the Korea Food and Drug Administration in 2007, remains the only commercialized CIK-based therapy for HCC. The agent received orphan drug designation from the U.S. FDA in 2018 for adjuvant treatment following curative therapy [149,150]. Despite these advances, the broader clinical application of CIK cell therapy is limited by challenges in large-scale cell production, standardization under good manufacturing practice (GMP) conditions, and inhibition of CIK cell activity by immunosuppressive factors such as myeloid-derived suppressor cells (MDSCs) [151].

### 3.3. Vaccines

Cancer vaccines are designed to initiate, enhance, or sustain antitumor immune responses by promoting immune recognition of TAAs or neoantigens [152]. As a therapeutic immunotherapy modality, cancer vaccines aim to elicit tumor-specific T-cell responses through several mechanisms, including de novo priming of naïve T cells against tumor antigens that fail to naturally induce immunity, amplification of pre-existing antitumor immune responses, and broadening of the repertoire of tumor-reactive T cells [153]. Through these mechanisms, cancer vaccines seek to enhance immune-mediated tumor control or regression.

Despite their conceptual appeal, the clinical translation of cancer vaccines has been hindered by several intrinsic challenges. Tumor antigen heterogeneity, immune tolerance, and the absence of universally shared TAAs significantly limit vaccine efficacy across patient populations [154]. In addition, vaccine-induced immune responses are often insufficient to overcome the profoundly TME characteristic of HCC. Consequently, therapeutic cancer vaccines have demonstrated limited clinical benefit when administered as monotherapies.

Unlike prophylactic vaccines, which are intended to prevent cancer development, therapeutic cancer vaccines are designed to strengthen immune responses against established malignancies, offering an alternative treatment strategy for patients with advanced disease [155]. In the context of HCC, therapeutic vaccination presents unique challenges due to chronic inflammation, immune exhaustion, and impaired antigen presentation within the liver. Nevertheless, advances in antigen discovery, vaccine delivery platforms, and immune adjuvant systems have renewed interest in vaccine-based immunotherapy for HCC.

Therapeutic cancer vaccines can be personalized according to the genetic and antigenic landscape of individual tumors, enabling more precise and patient-specific treatment approaches [156]. Based on their composition and delivery strategies, cancer vaccines under investigation for HCC can be broadly classified into peptide-based, viral vector-based, nucleic acid-based (DNA or RNA), cell-based, and neoantigen-based vaccines. Ongoing and completed clinical trials evaluating these vaccine platforms are summarized in Table 2.

#### 3.3.1. Peptide-Based Vaccines

Peptide-based vaccines utilize short amino acid sequences derived from TAAs that can be recognized by the immune system and presented to T cells via MHC molecules. Owing to their defined composition and antigen specificity, peptide-based vaccines offer advantages in terms of manufacturing feasibility, safety, and reduced risk of off-target toxicity or autoimmune reactions [157]. By inducing antigen-specific immune responses against TAAs expressed on cancer cells, peptide-based vaccines have shown therapeutic potential in HCC [158].

Typically, these vaccines consist of immunogenic peptide epitopes derived from well-characterized HCC-associated antigens, including AFP, GPC3, NY-ESO-1, epidermal growth factor receptor (EGFR), human epidermal growth factor receptor 2 (HER2), human telomerase reverse transcriptase (hTERT), and MAGE-A [159]. Multiple preclinical and early-phase clinical studies have evaluated peptide-based vaccination strategies targeting these antigens in HCC.

Among these targets, GPC3 has emerged as one of the most extensively investigated TAAs in HCC. In a phase I clinical trial, vaccination with GPC3-derived peptides elicited antigen-specific immune responses in patients with HCC without inducing severe treatment-related adverse events [160]. In another phase II study, a multi-peptide vaccine (GV1001) incorporating AFP, hTERT, and MAGE-A1 peptides was shown to reduce the frequency of immunosuppressive CD4^+^CD25^+^Foxp3^+^ regulatory T cells, while maintaining an acceptable safety profile [161]. In addition, a phase I/II clinical trial is currently evaluating an off-the-shelf multi-peptide HCC vaccine (IMA970A) in combination with the Toll-like receptor (TLR) 7/8 and retinoic acid-inducible gene I (RIG-I) agonist CV8102 as an immune adjuvant in patients with very early, early, and intermediate-stage HCC [92].

Despite these encouraging findings, the development of peptide-based vaccines faces several fundamental challenges. One major concern is the potential activation of autoreactive T cells due to the breaking of central or peripheral tolerance against self-derived TAAs, which may increase the risk of immune-related toxicity [162]. Additionally, low-level expression of certain TAAs in normal tissues raises the possibility of on-target off-tumor effects. Importantly, the complex pathophysiology of HCC and the presence of a highly immunosuppressive TME substantially limit the efficacy of peptide vaccines when administered as monotherapy [163].

Consequently, peptide-based vaccination alone is unlikely to achieve durable clinical benefit in HCC. To enhance therapeutic efficacy, current strategies increasingly emphasize combination approaches that integrate peptide-based vaccines with immune checkpoint inhibitors, immune adjuvants, or other immunomodulatory therapies. Such combinatorial regimens aim to augment antigen-specific T-cell priming, overcome immune tolerance, and counteract TME-mediated suppression, thereby improving clinical outcomes in patients with HCC [164].

#### 3.3.2. Dendritic Cell (DC) Vaccines

DC-based vaccines have attracted considerable attention as a cancer immunotherapy strategy owing to the central role of DCs as professional antigen-presenting cells capable of initiating and shaping antitumor immune responses [165]. DCs capture, process, and present tumor-derived antigens to T cells, thereby inducing antigen-specific cytotoxic and helper T-cell responses directed against malignant cells. In DC vaccine approaches, autologous or allogeneic DCs are generated ex vivo and loaded with TAAs, tumor lysates, or antigen-encoding nucleic acids, followed by reinfusion into patients. Upon administration, these antigen-loaded DCs migrate to secondary lymphoid organs, where they prime and activate tumor-reactive, antigen-specific T cells, thereby enhancing systemic antitumor immunity [166].

Several early-phase clinical studies have evaluated DC-based vaccines in HCC. In a phase I clinical trial (NCT01974661), a DC vaccine primed with AFP—a protein frequently overexpressed in HCC—was administered intratumorally as COMBIG-DC (ilixadencel), an allogeneic DC-based therapeutic vaccine. Immunological monitoring revealed evidence of vaccine-induced immune activation with a favorable safety profile and manageable adverse effects. In another phase I/IIa study, patients with HCC received mature DCs pulsed with recombinant fusion proteins derived from AFP, MAGE-1, and GPC3, resulting in the induction of tumor-specific immune responses and RFS [167].

More recently, advances in personalized immunotherapy have enabled the development of neoantigen-based DC vaccines. A personalized mRNA-loaded DC vaccine (LK101), administered in combination with tumor ablation, demonstrated enhanced immune responses and improved overall survival in patients with HCC, highlighting the potential of DC vaccines as a component of precision immunotherapy for liver cancer [168]. These findings suggest that DC-based vaccination may serve as an effective platform for inducing tumor-specific immunity, particularly when integrated with locoregional or systemic treatments.

Despite their therapeutic promise, several challenges limit the widespread clinical application of DC vaccines in HCC. Identifying highly immunogenic and tumor-specific antigens capable of eliciting robust and durable immune responses remains a major obstacle. In addition, inefficient migration of infused DCs to lymphoid tissues, functional impairment of DCs within the immunosuppressive TME, and tumor-mediated immune tolerance significantly constrain vaccine efficacy [169]. Ongoing research efforts are therefore focused on optimizing antigen selection, improving DC maturation and trafficking, and combining DC vaccines with immune checkpoint inhibitors or other immunomodulatory strategies to overcome immune suppression and enhance clinical outcomes.

#### 3.3.3. Viral Vector-Based Vaccines

Viral vector-based vaccines exploit the intrinsic ability of viruses to efficiently deliver genetic material into host cells and to induce strong innate and adaptive immune responses. Owing to their high transduction efficiency and immunostimulatory properties, viral vectors have been widely investigated as platforms for cancer vaccination by enabling effective presentation of TAAs or neoantigens to the immune system [170]. Antigen expression mediated by viral vectors promotes robust T-cell priming and enhances antigen-specific immune responses against malignant cells [171].

Several viral platforms, including adenoviruses, lentiviruses, retroviruses, and adeno-associated viruses (AAVs), have been evaluated for cancer vaccine development [172]. Each vector system exhibits distinct characteristics with respect to immunogenicity, tropism, genomic payload capacity, and safety, which critically influence their suitability for vaccine applications. Among these, adenoviral vectors have received particular attention due to their high transduction efficiency, relatively low pathogenicity, and capacity to accommodate large genetic inserts [173].

Preclinical and early-phase clinical studies have demonstrated the feasibility of viral vector-based vaccines in HCC. In a phase I clinical trial (NCT00844623), an adenoviral vector encoding herpes simplex virus thymidine kinase (HSV-TK), administered in combination with systemic ganciclovir, was evaluated in patients with HCC and showed acceptable safety and indications of improved survival [174]. In addition, personalized cancer vaccines employing modified adenoviral vectors encoding patient-specific neoantigens have been shown to elicit potent tumor-specific T-cell responses in preclinical and translational studies [175].

Despite these encouraging results, several challenges remain. Pre-existing antiviral immunity may limit transgene expression and vaccine efficacy, while excessive innate immune activation can impair sustained antigen presentation. Consequently, further optimization of vector design, dosing strategies, and combination with immune checkpoint inhibitors or other immunomodulatory approaches is required to fully realize the therapeutic potential of viral vector-based vaccines in HCC.

#### 3.3.4. DNA Vaccine

DNA vaccines represent a promising immunotherapeutic strategy for HCC due to their favorable safety profile, ease of manufacturing, and capacity to induce both humoral and cellular immune responses [176]. Following delivery of plasmid DNA encoding TAAs into host cells, the encoded antigen is endogenously expressed, processed, and presented via MHC molecules, thereby eliciting antigen-specific immune responses against tumor cells [177]. A phase I clinical trial (NCT01828762) evaluated the safety and immunogenicity of a DNA vaccine encoding GPC3, a TAA frequently overexpressed in HCC. The vaccine was well tolerated and did not induce significant treatment-related adverse events. In an ongoing phase I/IIa study, a personalized neoantigen DNA vaccine (GNOS-PV02) combined with an IL-12–expressing plasmid and pembrolizumab demonstrated a response rate of approximately 25% with manageable toxicity in patients with advanced HCC [178].

Despite these advances, DNA vaccines generally induce relatively weak immune responses when administered alone. Therefore, ongoing efforts focus on optimizing antigen design, improving delivery methods (e.g., electroporation or nanoparticle carriers), and incorporating immune adjuvants. Combination strategies integrating DNA vaccines with immune checkpoint inhibitors or locoregional therapies are expected to enhance immunogenicity and improve clinical outcomes in HCC.

#### 3.3.5. mRNA Vaccine

mRNA-based vaccines induce antitumor immune responses by directing host cells to transiently express tumor-associated antigens or immunomodulatory proteins. Advances in mRNA stabilization, lipid nanoparticle (LNP) delivery systems, and large-scale manufacturing have accelerated the development of mRNA vaccines for cancer immunotherapy, including HCC [179].

In a phase I clinical study, a small activating RNA (saRNA) targeting CCAAT/enhancer-binding protein alpha (CEBPA) was evaluated in patients with advanced HCC. Treatment with MTL-CEBPA demonstrated acceptable safety and synergistic activity when combined with tyrosine kinase inhibitors (NCT02716012) [180]. An ongoing phase I clinical trial is further evaluating MTL-CEBPA in combination with atezolizumab and bevacizumab in patients with advanced HCC [181]. In addition, preclinical studies using lipid nanoparticle–encapsulated mRNA encoding AFP have demonstrated robust AFP-specific CD8^+^ T-cell responses and protection against AFP-expressing HCC tumors in murine models [182]. Although mRNA-based vaccines for HCC remain in early stages of development, their flexibility, rapid manufacturability, and capacity for personalization make them an attractive platform. Continued clinical evaluation is required to establish long-term safety, efficacy, and optimal combination strategies with existing systemic therapies.

#### 3.3.6. Neoantigen Vaccines

Conventional cancer vaccines have largely relied on tumor lysates or shared TAAs such as AFP and GPC3; however, limited immunogenicity and poor tumor specificity have restricted their clinical efficacy. Consequently, enhancing antigen specificity and immunogenicity has become a central objective in cancer vaccine development [183].

Neoantigens are tumor-specific epitopes generated by somatic mutations, alternative splicing events, or post-translational modifications. Because neoantigens are absent from normal tissues and bypass central immune tolerance, they represent highly attractive targets for personalized cancer vaccination [184]. Neoantigen vaccines can be delivered using various platforms, including peptides, DNA, mRNA, dendritic cells, or viral vectors. HCC exhibits a moderate tumor mutational burden (TMB), averaging approximately two mutations per megabase, providing a limited but exploitable neoantigen repertoire.

Preclinical studies have identified immunogenic neoantigens capable of eliciting anti-tumor immune responses in HCC models [185,186]. However, early clinical studies have demonstrated modest efficacy. In one clinical investigation, neoantigen vaccination failed to significantly improve overall prognosis following curative treatment, although two out of ten patients remained relapse-free, with a median recurrence-free survival of 7.4 months [187]. Combination strategies have therefore emerged as a critical approach to enhance neoantigen vaccine efficacy. In preclinical HCC models, combining neoantigen vaccines with anti–PD-1 therapy resulted in robust antitumor responses, with up to 80% tumor regression and durable immune memory [188].

A phase I trial evaluating pembrolizumab in combination with a personalized neoantigen vaccine (GNOS-PV02) in advanced HCC is ongoing (NCT04251117). In an early interim analysis, partial responses were observed in 3 of the first 12 patients and stable disease in 5 patients; these findings should be interpreted cautiously given the exploratory nature and limited sample size typical of phase I studies [97]. These findings highlight the potential of immune checkpoint blockade to enhance the clinical efficacy of neoantigen-based vaccination in HCC. Neoantigen vaccines in combination with immune checkpoint inhibitors (ICIs) are also being evaluated in ongoing clinical trials for HCC, including regimens targeting the PD-1/PD-L1 axis (NCT05269381, NCT05761717, NCT04912765) and dual-checkpoint approaches targeting both CTLA-4 and PD-1/PD-L1 (NCT04248569).

Beyond immune checkpoint inhibition, additional strategies aimed at improving neoantigen vaccine efficacy focus on enhancing antigen delivery and presentation by antigen-presenting cells (APCs). Preclinical studies have demonstrated that red blood cell–mediated delivery of nanoparticle-encapsulated neoantigens to the spleen can selectively enhance antigen uptake and presentation by APCs [189]. Similarly, virus-like nanovaccines co-delivering neoantigens and Toll-like receptor (TLR) agonists, as well as DC-targeted delivery systems, have shown promise in augmenting antigen-specific immune responses [190]. In particular, virus-like silicon nanovaccines engineered to deliver both neoantigens and a TLR9 agonist to DCs have been shown to promote DC maturation, enhance antigen presentation, and induce robust CD8^+^ T-cell responses in preclinical models [191].

Neoantigen peptides can also be directly loaded onto DCs to generate DC-based vaccines. However, rapidly inducing a sufficient magnitude of tumor-specific T-cell responses remains challenging, and vaccine efficacy is often limited by T-cell exhaustion within the immunosuppressive tumor microenvironment [192]. Notably, a phase II clinical study demonstrated that combining neoantigen-loaded DC vaccines with ACT produced synergistic and complementary effects, providing patients with both an immediate supply of tumor-reactive effector T cells and long-term immunological memory. Patients who mounted measurable immune responses exhibited prolonged disease-free survival [193].

Intrinsic features of HCC further complicate the development of effective neoantigen vaccines. Each HCC tumor harbors an average of approximately 70 somatic mutations, of which computational algorithms predict that nearly 30% may generate candidate neoantigens [194]. However, only a small fraction (approximately 0.6–2%) of these predicted neoantigens are recognized by autologous T cells, indicating substantial limitations in current neoantigen prediction and selection strategies [195]. These observations suggest that many neoantigen vaccines developed using existing pipelines may lack truly immunogenic epitopes.

Several approaches are being explored to overcome these challenges, including the integration of radiotherapy to enhance neoantigen transcription and presentation, modulation of DNA mismatch repair pathways or RNA splicing to increase neoantigen diversity, and continued refinement of computational algorithms to improve neoantigen identification and prioritization [22]. Collectively, these strategies aim to maximize the immunogenic potential of neoantigen vaccines and improve their therapeutic impact in HCC.

### 3.4. Oncolytic Viruses (OVs)

OVs are replication-competent viruses that preferentially infect and replicate within tumor cells, leading to selective tumor cell lysis (oncolysis) while largely sparing normal tissues. In addition to direct cytolytic activity, OVs exert antitumor effects by initiating systemic antitumor immunity [196]. Virus-mediated tumor cell lysis results in the release of tumor antigens, danger-associated molecular patterns (DAMPs), and proinflammatory cytokines, including tumor necrosis factor-α (TNF-α) and type I interferons, thereby activating both innate and tumor-specific adaptive immune responses that contribute to sustained tumor eradication [197].

To date, a wide range of DNA and RNA viruses have been investigated as oncolytic platforms for cancer therapy, including vaccinia virus (VV), herpes simplex virus (HSV), adenovirus (AdV), vesicular stomatitis virus (VSV), measles virus (MV), parvovirus, reovirus, and Newcastle disease virus (NDV) [198]. A major milestone in the field of oncolytic virotherapy was the regulatory approval of talimogene laherparepvec (T-VEC; Imlygic™), a genetically engineered HSV-1 expressing granulocyte–macrophage colony-stimulating factor (GM-CSF), which demonstrated clinical efficacy in melanoma by enhancing dendritic cell maturation and antitumor immunity [199].

Oncolytic viral therapy represents a promising immunotherapeutic approach for HCC; however, only a limited number of OV platforms have progressed to advanced clinical evaluation in this disease. Pexastimogene devacirepvec (JX-594), a genetically engineered vaccinia virus, was granted orphan drug designation for HCC. JX-594 lacks the viral thymidine kinase (TK) gene, enabling selective replication in TK-expressing tumor cells, and is engineered to express GM-CSF and β-galactosidase to enhance antitumor immune responses. Notably, JX-594 can also infect tumor-associated vascular endothelial cells, leading to disruption of tumor vasculature and induction of tumor necrosis [200].

Intrahepatic administration of JX-594 demonstrated acceptable safety and dose-dependent improvements in overall survival (OS) in phase I (NCT00629759) and phase II (NCT00554372) clinical trials. However, subsequent studies failed to confirm a clear clinical benefit in patients with advanced HCC. JX-594 (pexastimogene devacirepvec; Pexa-Vec) did not improve outcomes either as second-line therapy after sorafenib failure or in combination with sorafenib in a phase III trial (NCT02562755). These negative results may, in part, reflect challenges related to treatment sequencing, patient selection, and/or potential pharmacologic or immunologic interactions between Pexa-Vec and sorafenib. Although first-line combination therapy with JX-594 and nivolumab achieved an objective response rate of 33.3% (NCT03071094), the study was discontinued due to safety concerns and the lack of efficacy observed in the pivotal PHOCUS and CheckMate-459 trials evaluating JX-594 and nivolumab, respectively.

Another oncolytic virus evaluated in HCC is Oncorine (H101), a genetically modified adenovirus type 5 lacking the E1B55K gene, which allows preferential replication in tumor cells with dysfunctional p53 signaling. In addition to inducing tumor-specific immune responses, intraperitoneal administration of H101 has shown encouraging activity in malignant ascites associated with multiple cancers, including HCC. A retrospective analysis of 476 patients demonstrated that combining H101 with transarterial chemoembolization (TACE) significantly prolonged OS and reduced tumor-related mortality in HCC [201]. The safety and efficacy of H101 in combination with tislelizumab and lenvatinib as a second-line therapy for HCC are currently being evaluated in an ongoing clinical trial (NCT05675462). In a phase I study (NCT00844623), intratumoral administration of a replication-defective adenovirus engineered to express herpes simplex virus thymidine kinase (HSV-TK), in combination with ganciclovir, was evaluated in patients with advanced HCC; the study reported stable disease in 60% of patients with an acceptable safety profile.

Despite their therapeutic promise, several challenges limit the clinical translation of OV-based therapies in HCC. These include inefficient delivery of viral particles to tumor sites, rapid antiviral immune clearance, and suppression of viral replication and immune activation by the immunosuppressive TME. Effective access of OVs to malignant cells remains a critical determinant of therapeutic success in solid tumors. To enhance OV efficacy, combination strategies incorporating epigenetic modulators, small-molecule inhibitors targeting oncogenic signaling pathways, or approaches that shift antiviral responses from type I to type II interferon signaling have been proposed [202]. In a phase II/III clinical trial (NCT00300521), adenovirus thymidine kinase (ADV-TK) gene therapy administered after liver transplantation was associated with a 54.8% three-year overall survival rate compared with the control group. In addition, an ongoing phase Ib/II study is evaluating intratumoral talimogene laherparepvec (T-VEC) in combination with systemic pembrolizumab (MK-3475; KEYNOTE-611) in HCC (NCT02509507).

Furthermore, next-generation recombinant OVs engineered to express therapeutic transgenes—such as proinflammatory cytokines, T-cell co-stimulatory molecules, or immune checkpoint inhibitors—represent a promising strategy to overcome TME-mediated immunosuppression and amplify antitumor immunity [203]. Collectively, these advances support the continued development of OV-based combination therapies as a critical component of future immunotherapeutic strategies for HCC.

### 3.5. Bispecific Antibodies (BsAbs)

Bispecific antibodies (BsAbs) are engineered molecules capable of simultaneously binding two distinct epitopes, thereby enabling coordinated modulation of immune responses or physical bridging between immune effector cells and tumor cells. Based on their molecular mode of action, BsAbs can be broadly classified into two functional categories: cis co-engagement, in which two targets on the same cell are simultaneously engaged (e.g., PD-1–CTLA-4 BsAbs that synergistically inhibit immune checkpoint signaling), and trans co-engagement, in which BsAbs bridge two different cell types (e.g., EpCAM–CD3 BsAbs that redirect T cells toward tumor cells) [204].

A defining advantage of BsAbs is their ability to spatially confine immune activation to the tumor microenvironment, thereby enhancing antitumor efficacy while minimizing systemic toxicity [205]. The first bispecific T-cell engager (BiTE) evaluated in HCC targeted EpCAM and CD3, demonstrating the feasibility of redirecting cytotoxic T cells toward EpCAM-expressing tumor cells [206]. In Europe, catumaxomab—an EpCAM-targeting trifunctional antibody—was approved for the management of malignant ascites, including those associated with HCC, highlighting the clinical relevance of EpCAM-directed strategies [207].

GPC3, a cell-surface proteoglycan selectively overexpressed in HCC, has emerged as another promising BsAb target. In xenograft mouse models, GPC3–CD3 BsAbs induced robust T-cell–mediated tumor regression, and their safety and preliminary efficacy have been evaluated in phase I clinical trials [208]. Beyond T-cell redirection, alternative BsAb designs have explored co-targeting GPC3 and immune regulatory molecules such as CD47. A GPC3–CD47 BsAb was shown to enhance Fc-mediated antitumor effects through macrophage- and neutrophil-dependent mechanisms, thereby integrating innate and adaptive immune effector functions [209].

BsAbs have also been engineered to target viral antigens expressed on HBV-associated HCC cells. In preclinical studies, BsAbs simultaneously recognizing hepatitis B virus envelope proteins (HBVenv) and CD3 or CD28 mediated potent cytotoxic and cytokine-driven antitumor responses against HBV-positive HCC cells [210]. These findings underscore the unique potential of BsAbs to exploit virus-associated tumor antigens in virally driven HCC.

Another important BsAb strategy involves dual immune checkpoint blockade. BsAbs targeting both PD-1 and CTLA-4 have been shown to selectively expand CD8^+^ PD-1 into tumor-infiltrating lymphocytes and induce tumor regression in HCC models. Notably, the PD-1–CTLA-4 BsAb MEDI5752 demonstrated enhanced immune activation with reduced systemic toxicity compared with conventional monoclonal antibody combinations [211]. Multiple clinical trials are currently evaluating PD-1–CTLA-4 BsAbs in advanced HCC, either as monotherapy (NCT04728321) or in combination with lenvatinib (NCT04444167), regorafenib (NCT05773105), or transarterial chemoembolization (TACE) (NCT05925413). Recently, a phase II open-label study (NCT04542837) evaluated KN046, a bispecific antibody targeting PD-L1 and CTLA-4, in combination with lenvatinib in advanced HCC, reporting a median PFS of 11 months and a median OS of 16.4 months. Another phase II study (NCT05775159) is assessing bispecific antibodies that simultaneously target two immune checkpoints on T cells (e.g., PD-1/CTLA-4 and PD-1/TIGIT), aiming to integrate the potential benefits of dual checkpoint blockade within a single molecule. Collectively, these studies position BsAbs as a versatile immunotherapeutic platform capable of integrating immune checkpoint modulation, tumor targeting, and effector cell recruitment in HCC.

### 3.6. Combination Immunotherapy Strategies

Despite substantial advances in immunotherapy for HCC, durable responses to single-agent immunotherapies remain limited. This is largely attributable to the intrinsic tolerogenic nature of the liver and the profoundly immunosuppressive TME characteristic of HCC. Historically, first-line systemic treatment for advanced HCC relied on multikinase inhibitors targeting angiogenesis and tumor growth pathways, including VEGFR, PDGFR, FGFR, BRAF, and c-KIT [212]. Subsequent treatment options included sorafenib or lenvatinib in the frontline setting, followed by regorafenib, cabozantinib, or ramucirumab after sorafenib failure [213].

Based on the therapeutic effect of certain ICIs and an improved insight of the TME, a number of combination methods can be explored and several of these currently are in clinical development.

An improved understanding of tumor immunobiology and TME-driven resistance mechanisms has catalyzed the development of rational combination therapies. Among these, immune checkpoint blockade (ICB) combined with anti-angiogenic therapy has emerged as a particularly effective strategy. After more than a decade of largely unsuccessful immunotherapy trials, two combination regimens demonstrated clear superiority over sorafenib. In the landmark IMbrave150 trial, patients were randomized to receive either sorafenib or atezolizumab (1200 mg) plus bevacizumab (15 mg/kg) every three weeks. The combination significantly improved overall survival (hazard ratio [HR] 0.58; 95% CI 0.42–0.79; *p* = 0.0006) and progression-free survival (HR 0.59; 95% CI 0.47–0.76; *p* < 0.0001), leading to early trial termination at the first interim analysis [55]. With extended follow-up, median overall survival reached 19.2 months in the combination arm versus 13.4 months with sorafenib, accompanied by superior response rates and preservation of health-related quality of life. Based on these results, regulatory agencies including the FDA and EMA approved atezolizumab plus bevacizumab as first-line therapy for advanced HCC.

Combination immune checkpoint blockade has also demonstrated clinical benefit. Dual inhibition of CTLA-4 and PD-1/PD-L1 increased objective response rates to approximately 30% in patients previously treated with sorafenib [214]. In particular, the combination of ipilimumab and nivolumab achieved a median overall survival of 22.8 months, leading to accelerated FDA approval for HCC patients who had progressed on sorafenib [215]. However, increased efficacy has frequently been accompanied by higher rates of immune-related adverse events, highlighting the need for careful patient selection and toxicity management. Similarly, combinations of ICIs with multikinase inhibitors such as cabozantinib and nivolumab have yielded improved response rates compared with monotherapy, albeit with increased toxicity [216].

Mechanistically, angiogenic signaling pathways contribute to immunosuppression by impairing APC function, inhibiting effector T-cell infiltration, and promoting the expansion of regulatory T cells and myeloid-derived suppressor cells [217]. Given that HCC is a highly vascular tumor characterized by elevated VEGF levels and an immunosuppressive TME, anti-angiogenic therapies may normalize tumor vasculature and enhance immune cell infiltration, thereby synergizing with immune checkpoint blockade [218].

In line with this rationale, pembrolizumab combined with lenvatinib was granted FDA breakthrough therapy designation for first-line treatment of patients with unresectable HCC not amenable to locoregional therapies, based on encouraging results from phase Ib studies [219]. Numerous ongoing clinical trials are further evaluating combinations of angiogenesis inhibitors with ICIs, as well as multi-modal regimens incorporating vaccines, oncolytic viruses, adoptive cell therapies, and BsAbs (Table 2). Collectively, these combination strategies represent the most promising avenue for overcoming immune resistance and achieving durable clinical benefit in HCC.

## 4. Potential Future Therapies

With multiple research avenues actively under investigation, the future of cancer immunotherapy for HCC remains promising. Advances in molecular characterization of HCC, together with efforts to identify predictive biomarkers for existing therapies, are progressively enabling the translation of recent discoveries into novel therapeutic targets, innovative treatment modalities, and precision biomarkers, with the ultimate goal of improving patient outcomes [220]. ICIs exemplify this progress, achieving objective response rates of approximately 19–20% with anti–PD-1 monotherapy, including complete responses in a small subset of patients and durable clinical benefit in selected cases [221]. Nevertheless, despite these advances, the overall impact on long-term survival at the population level has remained modest.

At present, immunotherapy is expected to remain the backbone of systemic treatment for advanced HCC until truly transformative therapeutic strategies emerge. Ongoing immunotherapy-driven clinical trials and rational combination regimens continue to shape the evolving HCC treatment landscape. As mechanistic insights into HCC progression and drug resistance deepen, precision medicine approaches—incorporating biomarker-guided patient stratification, rational sequencing of therapies, and synthetic lethal strategies—are likely to define the future trajectory of HCC treatment. Central to these approaches is sustained activation of the patient’s immune system, enabling cytotoxic T cells to recognize and eliminate tumor cells effectively.

Because immunosuppression is closely linked to treatment outcomes in HCC, strategies targeting immunosuppressive cell populations—particularly MDSCs—have been extensively investigated. These approaches include inhibiting MDSC expansion, blocking their recruitment and infiltration into the TME, suppressing their immunosuppressive functions, and promoting their differentiation and maturation. Several studies suggest that chemotherapy can reduce MDSC levels; however, its utility in HCC is often limited by chemoresistance and tolerability concerns in patients with underlying liver dysfunction. Accordingly, multiple clinical trials have evaluated combinations of chemotherapy and ICIs, aiming to enhance immune modulation and, in some settings, reduce MDSC burden. Overall, targeting MDSCs may offer therapeutic benefit given their multifaceted roles in shaping the TME. While MDSC-directed monotherapy has shown encouraging but generally limited efficacy, combination strategies—typically incorporating immunotherapy, kinase inhibitors, conventional chemotherapeutics, and/or all-trans retinoic acid (ATRA)—appear more effective, particularly when integrated with immunotherapy.

Currently, the most widely investigated immunotherapeutic regimens in HCC include combinations of ICIs with anti-VEGF/VEGFR agents, dual immune checkpoint blockade, and ICIs combined with kinase inhibitors (KIs) [222]. Among these, PD-1/PD-L1 and CTLA-4 dual blockade remains one of the most intensively studied strategies. Regulatory approvals now reflect this progress: the combination of tremelimumab (CTLA-4 inhibitor) and durvalumab (PD-L1 inhibitor) has been approved as first-line therapy, while nivolumab (PD-1 inhibitor) plus ipilimumab (CTLA-4 inhibitor) has been approved as second-line therapy for advanced HCC. Although some studies suggest that PD-1 inhibitors may yield superior median overall survival (mOS) and progression-free survival (mPFS) compared with PD-L1 inhibitors—owing to their ability to block both PD-L1 and PD-L2—additional head-to-head clinical trials are required to validate this hypothesis [223].

Angiogenesis represents another critical therapeutic axis in HCC. VEGF-driven aberrant vascularization contributes to immune exclusion and immunosuppression within the tumor microenvironment. Anti-VEGF/VEGFR therapies can normalize tumor vasculature, improve immune cell infiltration, and synergize with ICIs. Accordingly, atezolizumab plus bevacizumab has been approved by the FDA as first-line therapy for advanced HCC, while the National Medical Products Administration (NMPA) of China has approved sintilimab combined with IBI305 (a bevacizumab biosimilar) [224,225]. Recently, the National Medical Products Administration (NMPA) of China approved the combination of camrelizumab (a PD-1 inhibitor) and rivoceranib (a VEGFR inhibitor) as a first-line treatment for patients with advanced HCC [226]. In the phase III CARES-310 trial, this combination demonstrated statistically significant superiority over sorafenib in terms of both median overall survival (mOS) and median progression-free survival (mPFS), with mOS reaching 22.1 months [227]. These results underscore the therapeutic potential of combining immune checkpoint inhibitors with anti-VEGF/VEGFR agents and further support this strategy as a promising direction for first-line systemic therapy in advanced HCC.

Beyond established immune checkpoints, novel targets such as lymphocyte activation gene-3 (LAG-3) are gaining attention. LAG-3 is an inhibitory receptor that regulates T-cell activation and is frequently co-expressed with PD-1 on exhausted tumor-infiltrating lymphocytes. In HCC, LAG-3 expression has been detected on tumor-infiltrating CD8^+^ T cells in approximately 65% of resected tumors, with concurrent PD-L1 expression observed in over 80% of cases [228]. These findings support the rationale for next-generation immune checkpoint combinations incorporating LAG-3 blockade.

Kinase inhibitors (KIs) also continue to play a critical role in HCC therapy. Since the approval of lenvatinib in 2018, KIs have been recognized not only for their direct antitumor and antiangiogenic effects but also for their capacity to enhance tumor immunogenicity by promoting tumor necrosis and antigen release. Among KI–ICI combinations, lenvatinib plus pembrolizumab has been the most extensively studied. In the KEYNOTE-524 phase I trial, this combination achieved an objective response rate of 36%, approximately double that observed with pembrolizumab monotherapy. Other KI–ICI combinations, including axitinib and anlotinib-based regimens, have also demonstrated encouraging preliminary response rates approaching 30% [226].

Cellular therapies represent another frontier in HCC immunotherapy. Chimeric antigen receptor (CAR) T-cell therapies targeting AFP, GPC3, CD133, and c-MET are currently under clinical investigation. However, significant challenges remain, including insufficient trafficking and infiltration of CAR-T cells into solid tumors, antigen heterogeneity, and immunosuppressive signaling within the tumor microenvironment. Strategies under development include engineering CAR-T cells to express chemokine receptors to enhance tumor homing, local or regional delivery approaches, and combination regimens incorporating ICIs or other immunomodulatory agents to improve CAR-T cell persistence and function in HCC [229].

Cancer vaccines also represent a promising component of future HCC therapy. To address tumor heterogeneity, next-generation vaccines are increasingly personalized based on individual tumor antigenic landscapes. The identification of highly immunogenic tumor-associated antigens or neoantigens remains critical to vaccine efficacy. Successful vaccination strategies must overcome tumor-induced immunosuppression and enhance immune recognition and elimination of malignant cells [152]. Optimizing cancer vaccination strategies requires the integration of predictive biomarkers and robust analytical models. The identification of reliable biomarkers, together with the development of patient selection and response-monitoring frameworks, would enable more precise identification of patients most likely to benefit from vaccination-based therapies. Such approaches would also facilitate early detection of treatment response or resistance, thereby allowing timely therapeutic adaptation and more effective clinical decision-making. As our understanding of tumor biology and the immune system’s role in cancer progression continues to advance, the development of increasingly efficient and personalized cancer vaccines is becoming more feasible [230,231]. Future research should focus on the identification of novel immune adjuvants and immunomodulatory agents that can synergize with cancer vaccines to induce stronger and more durable antitumor immune responses. In parallel, advances in antigen delivery platforms, including lipid nanoparticles and viral vectors, are expected to enhance vaccine potency and targeting accuracy, ultimately translating into improved therapeutic efficacy [232].

Ultimately, progress in HCC immunotherapy will depend on close collaboration among basic scientists, clinicians, industry partners, and regulatory agencies. Improved preclinical models, refined clinical trial designs, and integration of real-world evidence will be essential to evaluate long-term safety and efficacy across diverse patient populations. By addressing current limitations and leveraging emerging opportunities, these collaborative efforts may substantially improve both survival and quality of life for patients with HCC.

## 5. Conclusions

In summary, immunotherapeutic strategies for hepatocellular carcinoma have advanced rapidly in recent years, offering new opportunities to overcome longstanding therapeutic limitations. Nevertheless, several critical challenges remain, including the paucity of highly druggable oncogenic targets, the lack of robust molecular biomarkers for patient stratification and treatment guidance, and the absence of universally accepted algorithms for optimal therapy selection. These issues are particularly relevant in HCC, where underlying liver dysfunction further complicates treatment decisions.

Future research must balance broad access to innovative therapies with careful patient selection, especially for individuals with compromised or borderline hepatic reserve who may still derive meaningful clinical benefit from immunotherapy. Rational integration of biomarkers, combination regimens, and adaptive treatment strategies will be essential to maximize efficacy while minimizing toxicity.

Overall, the immunotherapy landscape for HCC is evolving toward integrated, multi-platform approaches that combine immune checkpoint blockade, anti-angiogenic therapy, cellular therapies, oncolytic viruses, and cancer vaccines. Continued collaboration among academia, industry, and regulatory authorities will be critical to accelerate the development, validation, and approval of next-generation immunotherapies. If these efforts succeed, the therapeutic paradigm for HCC may undergo a fundamental transformation, ultimately translating scientific advances into durable clinical benefit for patients.

## Figures and Tables

**Table 1:** Approved Immunotherapies for HCC.

Drug Name	Trial Name	Commercial Name	Trial Phase	Approval Year	Mode of Action	Median PFS/Median OS (Months)	Trial Number	Ref.
Sorafenib	SHARP	Nexavar	III	2007	Multikinase inhibitor; inhibits tumor cell proliferation and angiogenesis	5.5/10.7	NCT00105443	[52]
Lenvatinib	REFLECT	LENVIMA	III	2018	Multikinase inhibitor; anti-angiogenic and antiproliferative activity	7.4/13.6	NCT01761266	[53]
Atezolizumab + bevacizumab	IMbrave150	Tecentriq & Avastin	III	2020	PD-L1 blockade combined with VEGF inhibition	6.8/19.2	NCT03434379	[54,55]
Regorafenib	RESORCE	Stivarga	III	2017	Multikinase inhibitor; TME modulation and anti-metastatic activity	3.1/10.6	NCT01774344	[56]
Cabozantinib	CELESTIAL	Cabometyx	III	2019	Multikinase inhibitor targeting angiogenesis and tumor growth	5.2/10.2	NCT01908426	[57]
Ramucirumab	REACH-2	Cyramza	III	2019	VEGFR-2 monoclonal antibody; anti-angiogenic	2.8/8.5	NCT02435433	[58]
Nivolumab + ipilimumab	CheckMate 9DW	Opdivo	III	2025	Dual immune checkpoint blockade (PD-1 + CTLA-4)	9.1/23.7	NCT04039607	[59]
Pembrolizumab	KEYNOTE-394	Keytruda	III	2018	PD-1 immune checkpoint inhibition	2.6/14.6	NCT03062358	[60]
Ramucirumab	REACH-2	Cyramza	III	2019	VEGFR-2 monoclonal antibody; anti-angiogenic	2.8/8.5	NCT02435433	[61]

**Table 2:** Selected Completed and Ongoing Clinical Trials of Immunotherapy-Based Strategies for HCC.

Type	Drugs	Trial Phase	Trial Name	Status	Targets	Combination	Trial Number	Ref.
Immune Checkpoint Inhibitors (ICIs)	Nivolumab	I/II	CheckMate040	Completed	PD-1	Ipilimumab & Cabozantinib	NCT01658878	[62]
	Tremelimumab	II	-	Completed	CTLA-4	-	NCT01008358	[63]
	Avelumab	II	AvelumabHCC	Completed	PD-L1	-	NCT03389126	[64]
	Tislelizumab	II	RATIONALE-208	Completed	PD-1	-	NCT03419897	[65]
	Nivolumab	II	-	Completed	PD-1/CTLA-4	Ipilimumab	NCT03510871	[66]
	Nivolumab	III	CheckMate 459	Completed	PD-1	-	NCT02576509	[67]
	Atezolizumab	III	IMbrave150	Completed	PD-L1	Bevacizumab	NCT03434379	[68]
	Lenvatinib	III	LEAP-002	Completed	VEGFRs	Pembrolizumab	NCT03713593	[69]
	Sorafenib	II	-	Completed	VEGF/PDGFR	Nivolumab	NCT03439891	[70]
	Pembrolizumab	III	MK-3475-937/KEYNOTE-937	Ongoing	PD-1		NCT03867084	[71]
	Durvalumab	III	EMERALD-2	Ongoing	PD-L1	Monotherapy & bevacizumab	NCT03847428	[72]
	Durvalumab	III	HIMALAYA	Ongoing	PD-L2	Tremelimumab	NCT03298451	[73]
	Nivolumab	III	CheckMate 9DX	Ongoing	PD-1	_	NCT03383458	[74]
	Cabozantinib	III	COSMIC-312	Ongoing	VEGFRs	Atezolizumab	NCT03755791	[75]
	Atezolizumab	III	IMbrave251	Ongoing	PD-L1	Lenvatinib/Sorafenib	NCT04770896	[76]
	Finotonlimab (SCT-I10A)	II/III	-	Ongoing	PD-L1	Bevacizumab (SCT510)	NCT04560894	[77]
Adoptive Cell Therapy (ACT)	CAR-T Cell Therapy							
	GPC3	I	GLYCAR	Completed	GPC3^+^ tumor cells		NCT02905188	[78]
	GPC3	Pilot	-	Completed	GPC3^+^ tumor cells		NCT03146234	[79]
	GPC3	I	-	Completed	GPC3^+^ tumor cells		NCT02395250	[80]
	GPC3	I	-	Completed	GPC3^+^ tumor cells		NCT03980288	[81]
	GPC3	I	-	Completed	GPC3^+^ tumor cells		NCT03884751	[82]
	B7H3	I/II	-	Ongoing	B7-H3^+^ tumor cells		NCT05323201	[83]
	GPC3	I	-	Ongoing	GPC3^+^ tumor cells		NCT05003895	[84]
	NK/CIK Cell Therapy							
	NK cells + IRE	I/II	-	Completed			NCT03008343	[85]
	NK cell therapy	I	MIAMINK	Completed			NCT01147380	[86]
	NK cell therapy	II	MG4101	Completed			NCT02008929	[87]
	Cytokine-induced Killer (CIK) Cell	III	-	Completed			NCT01749865	[88]
	Immuncell-LC (CIK)	III	-	Completed			NCT00699816	[89]
	CIK Cell	III	HCC-CIK	Completed			NCT00769106	[90]
Vaccines and OV-based Therapies								
Vaccine (DC)	COMBIG-DC	I	-	Completed		-	NCT01974661	[91]
Vaccine (Peptide)	IMA970A	I/II	HepaVac-101	Completed		CV8102	NCT03203005	[92]
OV	Pexa-Vec (JX-594)	III	PHOCUS	Completed		Sorafenib	NCT02562755	[93]
Vaccine (DC-TC)	DC-TC	Pilot	-	Completed		-	NCT01828762	[94]
Vaccine (Peptide)	DNAJB1-PRKACA	I	-	Ongoing		Nivolumab + Ipilimumab	NCT04248569	[95]
Vaccine (Neo-DC)	Neoantigen DC Vaccine	II	-	Ongoing		-	NCT04912765	[96]
Vaccine (Neoantigen)	GNOS-PV02 + INO-9012	I/II	-	Ongoing		Pembrolizumab	NCT04251117	[97]
Vaccine (Neo-peptide)	PNeoVCA	I/II	PNeoVCA	Ongoing		Pembrolizumab	NCT05269381	[98]
Vaccine (Fusion peptide)	FusionVAC22	I	FusionVAC22	Ongoing		Atezolizumab	NCT05937295	[99]

## Data Availability

The data of this study are available from the corresponding author on reasonable request.
